# eFarm: A Tool for Better Observing Agricultural Land Systems

**DOI:** 10.3390/s17030453

**Published:** 2017-02-24

**Authors:** Qiangyi Yu, Yun Shi, Huajun Tang, Peng Yang, Ankun Xie, Bin Liu, Wenbin Wu

**Affiliations:** Key Laboratory of Agricultural Remote Sensing (AGRIRS), Ministry of Agriculture/Institute of Agricultural Resources and Regional Planning, Chinese Academy of Agricultural Sciences, Beijing 100081, China; yuqiangyi@caas.cn (Q.Y.); tanghuajun@caas.cn (H.T.); yangpeng@caas.cn (P.Y.); xieankun@caas.cn (A.X.); liubin.caas@foxmail.com (B.L.)

**Keywords:** smartphone, human sensing, social sensing, crowdsourcing, agriculture, land use, citizen science

## Abstract

Currently, observations of an agricultural land system (ALS) largely depend on remotely-sensed images, focusing on its biophysical features. While social surveys capture the socioeconomic features, the information was inadequately integrated with the biophysical features of an ALS and the applications are limited due to the issues of cost and efficiency to carry out such detailed and comparable social surveys at a large spatial coverage. In this paper, we introduce a smartphone-based app, called eFarm: a crowdsourcing and human sensing tool to collect the geotagged ALS information at the land parcel level, based on the high resolution remotely-sensed images. We illustrate its main functionalities, including map visualization, data management, and data sensing. Results of the trial test suggest the system works well. We believe the tool is able to acquire the human–land integrated information which is broadly-covered and timely-updated, thus presenting great potential for improving sensing, mapping, and modeling of ALS studies.

## 1. Introduction

Land constitutes the terrestrial component of the earth and stands at the center of the coupled human and natural systems [1], while agricultural land occupies about 38% of earth’s terrestrial surface: the largest use of land on the planet [2]. Land systems reflect the state and result of human systems interacting with the natural system, while the agricultural land system (ALS)—with the functionality of providing vital resources to society, such as food, fuel, fibers and many other ecosystem services that support production—is one of the most important land systems for human society [3]. Understanding the spatial-temporal characteristics of agricultural area, its internal states such as crop allocation, farm management, and disturbance, as well as the human activities that are relevant to the changes of the state, have great implications on food security, sustainability, and social development [3,4,5].

The biophysical features of ALS have been effectively sensed by remote sensing techniques, which covers a wide range of observation from land cover (i.e., cropland distribution) to crop parameters (e.g., crop phenology, biomass, and yield), as well as the estimation of soil moisture and drought [6,7]. On one hand, there are a number of existing regional/global land cover datasets derived from remotely-sensed images, which are able to provide information on cropland distribution and its changes in both spatial and temporal dimensions [8,9]. In many cases, the preliminary remotely-sensed images received from satellites—which have not been processed to land cover data yet—have been used directly to observe the state and changes of cropland, giving particular attention to some hot-spot areas [10]. These observations help to understand land cover though land use and farm management, which change more frequently without any significant change of the land cover, is more difficult to observe [3]. It implies that untimely cropland information is insufficient to support the multi-faceted ALS studies. On the other hand, some studies attempt to use remote sensing for partly investigating agricultural intensification and farm management rather than the mere attention paid to the cropland mask. For example, Li, et al. [11] mapped the cropland growth stages (e.g., dates of onset/peak/end of growth, and the length of the growing season) in northeast China by using the SPOT-VGT time-series data. This type of study promotes the remote sensing application from qualitative classification to quantitative monitoring that are able to reflect more information of human activities. However, given that the focus of remote sensing is still the physical objectives on land surface, the integration of biophysical and socioeconomic features for agricultural land system observation is inadequate. Consequently, one limitation of Li, et al. [11] was that the results are somehow difficult to link with real stages of crop growth such as sown, maturity, and harvest to reflect the real activities of farm management.

From the socioeconomic perspective, a number of studies have tried to understand ALS using surveys and statistics data [12,13,14]. However, the linkages between human and land were usually weak. For example, social surveys are able to acquire human land use activities while few of them were interested in the location of land and the spatial allocation of land use activities. Moreover, the statistics are only able to represent the aggregated land use and its related human activities at an administrative level. This results in difficulties such as explaining how the decisions made by land managers would change the state of a land parcel and how the changes on land were determined by land managers. Some other studies combined household surveys with remote sensing by providing maps to interviewees to acquire information spatially. These surveys commonly focused on human sentiments toward land use, thus they are good at analyzing the consequences or causes of ALS change [15,16] rather than the observation itself. A pilot study from Yu, et al. [17] tried to map the combined biophysical and socioeconomic features of a local level ALS based on a well-designed household survey. However, the applications of such an approach are extremely limited, because remote sensing is able to capture large scale information though, field surveys are always limited to finer scales with huge expense on time, money, and human recourses. Moreover, the traditional household surveys require respondents to recall the past events, which would lead to a higher data unreliability when the recall period was long [18]. All these suggest that the traditional survey approaches have limited potential in observing agricultural land systems.

Given the nature of agriculture–i.e., underpinned by the biophysical environment while affected by human activities–a better sensing of ALS needs to integrate both its biophysical and socioeconomic characteristics. Moreover, its rapid changes due to crop growth and disturbance require the timely observation at a broad spatial coverage and the ability to elicit the heterogeneity at specific sites to inform better management. Unfortunately, no existing reports or tools were able to address all these aspects in the observations on ALS. Since the popularization of the internet and advanced data storage and computing technology, there has been an explosion of interest in using the web to collect, assemble, and disseminate information, which stimulated the development of citizen science in the latest decade [19,20,21]. In this paper, we introduce a recently-developed tool based on the idea of citizen science, which is able to revolutionize the observation for improving ALS studies.

## 2. Improving ALS Observation Based on Existing Sensing Technologies

**Figure 1:**
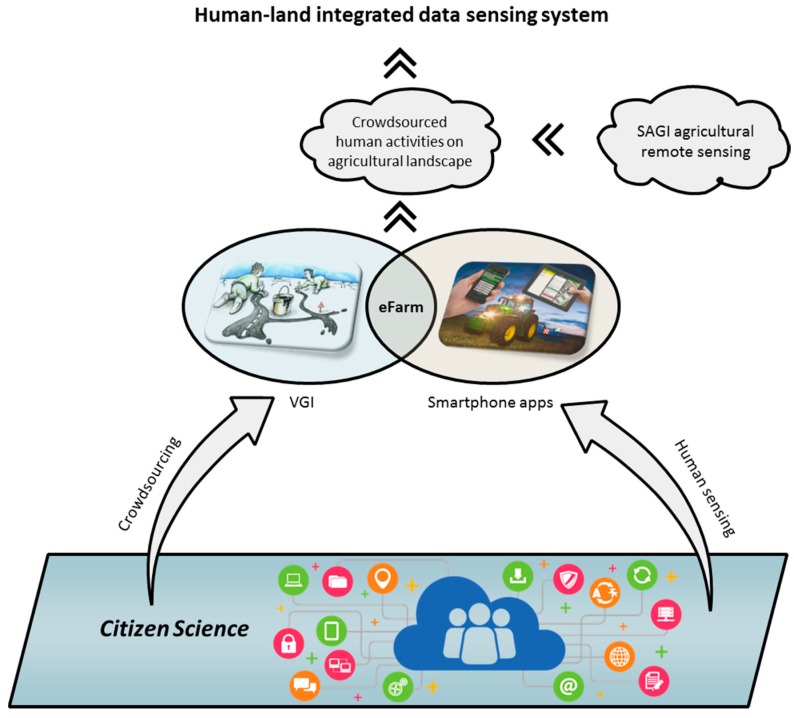
The concept of improving observation of ALS using support from relevant disciplines. Based on agricultural remote sensing, new sensing techniques from citizen science such as crowdsourcing and human sensing are applied to expand the potential of traditional household surveys in acquiring the human-land integrated information. Abbreviations in the figure: VGI: Volunteered Geographic Information; SAGI: Satellite, Aerial, and Ground Integrated agricultural remote sensing. The details of the concepts are elaborated in this section below. Some elements of the figure are adopted from internet.

In order to better observe ALS, spatial-temporal characteristics need to be captured timely with a broad spatial coverage, and to be able to integrate the farmer and their land to better reflect human-environment interactions. There are a few existing technologies or platforms available in the relevant disciplines. Although they are indirectly/less related to the observation of ALS, combing their unique advantages stands a chance at addressing all these requirements together. This stimulates the development of the new sensing tool for improving the observation of ALS (Figure 1).

### 2.1. SAGI Agricultural Remote Sensing

Although satellite imagery has been playing an important role in agricultural remote sensing, limitations exist that they may not be able to supply sufficient information with adequate resolution, accurate geo-referencing, and specialized biological parameters. A more advanced platform for agricultural remote sensing, known as SAGI (Satellite, Aerial, and Ground Integrated agricultural remote sensing) has been developed and implemented by the authors’ host institute: Institute of Agricultural Resources and Regional Planning, Chinese Academy of Agricultural Sciences (IARRP, CAAS) to enforce a stronger information integration with respect to joint data processing, image sequence registration, and data assimilation [22]. Although SAGI proposed a solution to data harmonization that substantially improves observation, it still focuses on the biophysical characteristics of agricultural land.

### 2.2. Smartphone Sensing in Agriculture

In pace of the prevalent application of smartphone sensors in industries such as education, health care, public communication, and transportation [23,24,25], the usage in agricultural sector falls a little bit behind (e.g., the relevant publications and applications can only be seen in recent years) [26,27]. However, it is no doubt that the usefulness, ease-of-use, and affordability of built-in sensors have unlimited possibility to help the transformation of traditional agriculture [26]. Advantages through the access of cameras, microphones and recording software, geographical information, and global positioning systems (GPS), allow a variety of practical applications to be created, in both farming and farm management [26,27].

Currently, the smartphone applications in agriculture mainly focus on specific farming activities, such as water and fertilizer calculation, disease detection and diagnosis, pest and weed control, chlorophyll content estimation [28], and tractor-navigation, etc. The available apps which are ready to use include MyPestGuide (https://mypestguide.agric.wa.gov.au/), Di@gnoPlant (http://www.inra.fr/), WISE (Water Irrigation Scheduling for Efficient Application) [29], Cotton SmartIrrigation App [30], LCFSS (land consolidation field survey system) [31], among others. Compared with specific farming activities, the general and comprehensive applications on farmland management are relatively rare [26]. By using big data on three explanatory variables (household size, number of livestock, and land area) across the African continent, Frelat, et al. [32] suggested that macro level solutions such as improving market access to diversifying employment sources would have better consequences on poverty reduction and food security, rather than focusing on farming and closing yield gaps at the field level. It implies that application of smartphone sensing may play a more important role, if the priority is given to big data collection on farmland management.

### 2.3. Volunteered Geographic Information (VGI)

Goodchild [33] proposed the term VGI and defined it as user-generated content in geography. A number of technologies made VGI not only possible at the theory level but also practical at the application level. These enabling technologies include web 2.0, georeferencing, geotags, graphics, GPS, and broadband communication. Volunteers have been involved in sites such as Wikimapia, OpenStreetMap, and Geo-Wiki, by either creating a global patchwork of geographic information or developing interesting applications based on existing data. The main purpose of these tools differ from each other, i.e., Wikimapia is focused on place descriptions (www.wikimapia.org/), OpenStreetMap creates user-editable maps [34], and Geo-Wiki collects ground truth to validate global land cover datasets [35,36]. However, by searching the words of “volunteered geographic information” and “agriculture” at Google Scholar, it suggests that VGI has been rarely applied for agricultural purposes (e.g., farmland management), despite site-specification and georeference also being the nature of agriculture [33].

### 2.4. Crowdsourcing and Human Sensing

Crowdsourcing and human sensing are two commonly used concepts siting at the center of citizen science [37,38]. One of the significant characteristics of citizen science is the wide coverage of participation of people from the public. It is based on the involvement of a large number of volunteers in the research process, mainly during the data-collection stage [39]. This phenomenon is referred as ‘crowdsourcing’, represented by the success of VGI [33]. The other characteristic is that, with the help of modern sensors, e.g., sensors embedded in a smartphone which mainly reflect human activities, citizen science is able to expand the coverage of data content that are useful to advance the understanding of environmental science or human-environmental interactions from a human-centric perspective [24,25,40]. This methodology can be conceptualized as ‘human-sensing’. Combining these two advantages, ALS would be better understood if crowdsourcing is applied to expand the coverage of field survey and human-sensing is applied to integrate human activities into the observation of the biophysical state of agricultural landscapes.

## 3. The Development of eFarm

### 3.1. System Overview

**Figure 2:**
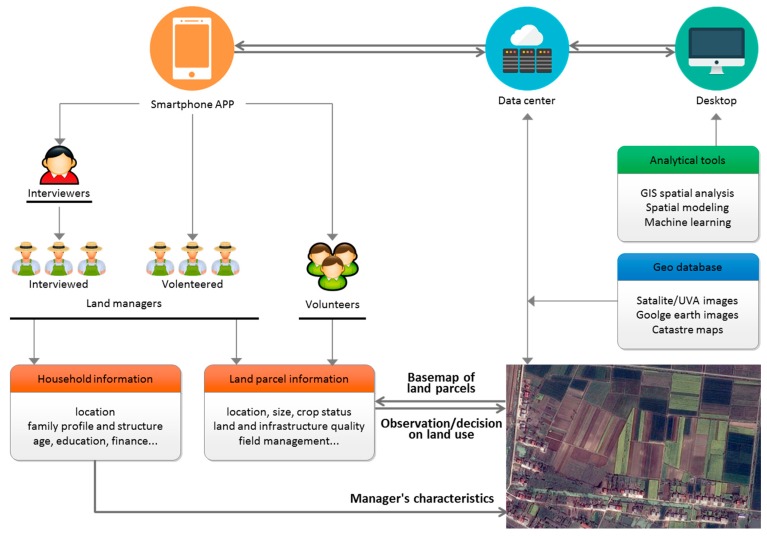
The overview of the eFarm system. The diagram presents a closed loop of information sensing: remoted-sensed images provide a basemap of land parcel information while the observed land use information and manager’s characteristics are added to the land parcels thought eFarm. The illustrated basemap was adopted from Google Map displaying an agricultural area in Qianjiang City, Central China. See a color-blinded figure in the Supplementary Materials.

The requirements of improving ALS observation and progress in the relevant disciplines inspired the development of eFarm, which is designed as a human-land integrated data sensing system represented by a data collecting app installed in smartphones. It combines agriculture and geography and allows volunteers to contribute the timely and georeferenced farmland management information to be integrated to the spatial land parcels derived from high resolution remotely-sensed images (Figure 1). The overview of the eFarm system is presented in Figure 2: the orange labeled section illustrates how the smartphone app works; the blue labeled section illustrates how data is exchanged among different platforms; and the green labeled section shows the how the comprehensive ALS information will be applied for sophisticated analysis. The app was originally developed based on the Android tablet system. Its main functionalities include visualization (of basemaps), data management (of land parcels and users), and data sensing (of land and household information). Detailed elaborations on the functionalities are described below.

### 3.2. Visualization of Basemaps

**Figure 3:**
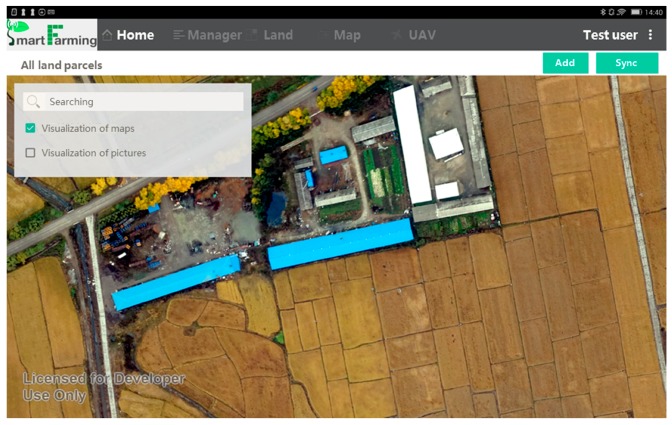
Visualization of a basemap in the eFarm app based on a timely acquired UAV image.

In order to observe the human-land integrated information on agricultural landscape, it is necessary to present high resolution, clearly visible, and georeferenced images as basemaps which are able to be referred and edited on the app. The Application Programming Interface (API) enables external maps and images—from a third party such as Google Map (https://maps.google.com/), Baidu Map (http://map.baidu.com/), and Tianditu (http://www.tianditu.com/)—to be visualized in the system for a particular region as the basemap (Figure 3). In addition, the basemap could be the timely acquired UAV (unmanned aerial vehicle) images from the SAGI agricultural remote sensing system when other images are insufficient (Figure 3). The candidate basemaps are prepared in the data center (e.g., selection and geometric calibration) before they are visualized in the smartphone app, and the maps with unclear boundaries of land parcels are excluded at this prescreening stage. The well-prepared maps in the data center can be freely switched, displayed and zoomed-in/out when they are ready to be viewed in the app with internet. Visualized maps will be automatically downloaded locally, so that the internet is no longer required for further visualization. The specific meta information (e.g., the exact data acquiring time, and the spatial extent) is accessible associated with the visualized map, and the default visualized map is given to the latest captured one.

### 3.3. Management of Land Parcels

**Figure 4:**
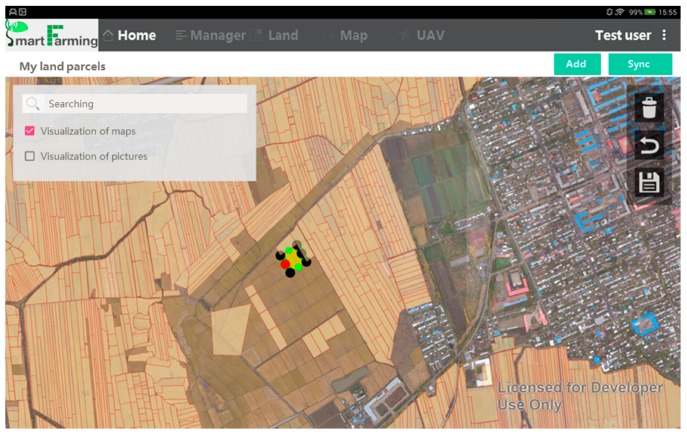
Creating a land parcel polygon based on a manual-drawing process.

Land parcels are the basic spatial units managed in the system and the foundation that sustains the human-land integrated information. They are identified based on a basemap and representing a unified farm management inside (while the management might be different among each other). A layer of land parcels will be created and stored in the geodatabase by looking at a basemap as the spatial information reference. Any independent land parcel in the shape of a polygon needs to be created by users one by one: it can be done by either a free-selecting tool to or a manual-drawing tool (Figure 4). The free-selecting is similar to the polygonal lasso, which is applicable when the size, location, and boundaries of land parcels are clearly identified. The algorithm of the lasso is developed in the SAGI system and embedded into the app [22]. The manually-drawing is similar to the process of creating a closed polygon on top of an area of the image, which starts by tapping at the starting point for the polygon, then moving the cursor to the next point of the polygon. A closed polygon representing the shape of an independent land parcel will be created when double-tapping at the last point. It is a substitute for the free selection when the lasso fails. The physical features of a land parcel (e.g., X-Y location, size, elevation, etc.) will be automatically captured after it has been created. Topological relationship will be checked in order to mark out the overlapped polygons. Considering the physical shape of land parcel might be changing, the geodatabase is managed on a yearly basis. It means overlapping might exist when the polygons are representing the state of different years.

### 3.4. Management of Users

The registered users are the non-spatial units managed in the system and the crowdsourcing sensors help to observe the state of ALS. There are three groups of users defined in the system: land managers, interviewers, and volunteers (Figure 2):

1. Land managers are the most important users, because they are the ultimate decision-maker in ALS and their land use activities will directly affect the state of the land parcel managed by themselves. Land managers could be either interviewed or volunteered. Thus, the interviewers are consisting the second group of user, who are responsible for organizing and conducting interviews toward land managers.
2. Interviewers are also important, given the literacy and incentive might not be sufficient enough for rural land managers to voluntarily report their land use activities. Moreover, the information from interviewed land managers are supposed to be more reliable than from the volunteered land managers. Each interviewer can have the relation with multiple land managers, while each land manager can manage multiple land parcels. Ideally, the interview processes are similar to the traditional household surveys, and the interviewer users should be scientific researchers who are involved in collecting and using the data in relevant researching programs. It is hoped that the systems can be operated as the LTER (Long Term Ecological Research Network), which is attracting many researchers and shifting focus from site-specific observations to a broader synthetic view aimed at searching out general principles that apply to many ALS at many different scales.
3. In addition to interviewers and land managers, the third user group is volunteers who are willing to contribute their witnessed land use information on land parcels. However, as they are not land managers, some information is not required (e.g., the household characteristics). The setup of volunteer users expands the number of sensors that would further enlarge the coverage of crowdsourcing. For example, it will make a better involvement of scientific researchers in a form that they are able to contribute real observing results rather than organizing household surveys.

Each registered user will be given a unique ID, which will be used to build the linkage between interviewer and interviewed land manager as well as the linkage between human sensor (i.e., land manager and volunteer) to the observed land parcels. For implementing a more efficient user management, any registered user of land, manager, or interviewer can be automatically activated as a volunteer user, when they are coding the land parcel not owned by themselves or without knowing the operational owner of the land.

### 3.5. Sensing of Land Information

Data sensing is followed by the visualization of maps and the development of data management units. This is a method of basic field information collection in use already—e.g., OpenStreetMap and Geo-Wiki both have smartphone apps—and the map component and smart phone link is promising. With unique purposes of data sensing, eFarm aims to identify the location of a land parcel and to acquire the additional information of the land parcel ranging from ownership to farmland management and to manager’s characteristics. Specifically, the data sensing procedure falls into two steps: spatial data management unit (i.e., sensing of land information, elaborated in this section below) and non-spatial data management unit (i.e., collecting household information, see Section 3.6).

**Figure 5:**
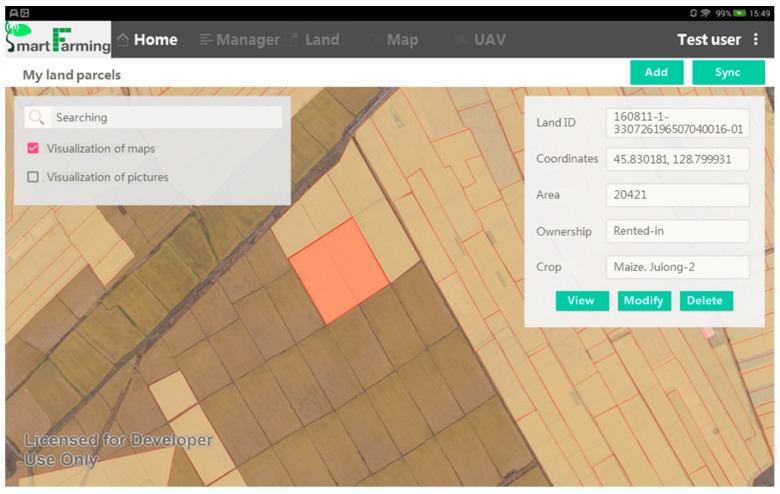
Visualization of land activities on a land parcel.

For the spatial data management unit, the land use activities observed/recorded by registered users will be coded to each land parcel to finalize the human sensing of human–land integrated information. The land parcel ID contains three parts, representing how the land parcel is linked with the observer: the first part records the time of observation (e.g., yymmdd), the intermediate part indicates the identity of the human sensor (1 means interviewed land manager, 2 means volunteered land manager, and 3 means volunteer), and the final part is the ID of the user. This coding scheme is not only useful for linking land managers with their managed land parcels, but also for quality control during the crowdsourcing process, especially when a land parcel is multiply recorded. It is supposed that the data quality is following a descending order from interviewed land managers to volunteered land managers to volunteers. The basic information required for the newly-created land parcels includes land ownership and management rights, land quality, crop choice, crop variety, fertilization, management calendar, and production (Figure 2). All of this land use information in addition with the physical features of the land parcel (e.g., X-Y location, size, elevation, etc.) can be visualized in the app, in associating with the visualization of the basemaps (Figure 5).

In addition to the traditional information inputting approach, ancillary information sensed from the embedded smartphone sensors such as GPS and camera will also be applied for the observation of land use activates. GPS is mostly used for location-aware applications. For example, when the app is opened, it will start searching and displaying the surrounding maps within a buffer zone centered by the location of the smartphone (the visualization center can be freely reallocated when dragging the screen). Moreover, it will receive essential environmental information including the location of land parcels, location of houses and location of land managers, and capture the movement of land managers when they go to (or work at) their fields. Such trajectories could be further processed in the desktop systems to analyze the fragmentation and intensity of agricultural land from a labor-input perspective.

Smartphone cameras are important in taking pictures and videos for reflecting the reality or for further image processing. The camera can be used in some occasions, for example, taking real-time pictures for the identified land parcels. This is particularly helpful because it is able to provide a sense of the field that is more straightforward and reliable. Furthermore, the pictures will be taken associating with other embedded sensors such as accelerometer, gyroscope, and magnetometer to facilitate the 3-D modeling and visualization of land parcels, which will be further integrated into the SAGI remote sensing system. The cameras will also be used to capture pictures instead of inputting the required information by words. For example, the personal identity information will be automatically retrieved based on taking a picture of the ID card. Moreover, the detailed information of used agricultural inputs such as seed and fertilizer can be acquired by taking pictures on the packing bags.

### 3.6. Collecting Household Information

For the non-spatial data management unit, household characteristics are required when linking the land parcel with its manager. This would expand the dimension of observation on ALS as the manager’s characteristics are integrated. Land managers need to fill such additional information at the registration stage, while it is not necessary for the other two groups of users, because land parcel information provided by volunteers can be stored and processed without knowing its owner. The basic household characteristics include household head’s age and education, household profile, and finances etc. (Figure 2). This information would be associated with the land parcels through the ID linkages. Moreover, the linkage between the interviewers and their interviewed land managers will be created to further improve the data quality control.

There is an additional function reserved for collecting the human sensing data: the survey questionnaires for both aspects of land use activated and household activities can be changed, edited, or redesigned according to specific research objectives. For example, the price, brand, and amount of consumed fertilizer, as well as the average annual production, disasters, and market price, etc. can be designed into a form for the land managers to fill. All of the extra information will be linked to the specific land parcel IDs directly or through the land manager ID. Moreover, once the design of questionnaires is finished at the desktop system, the table can be viewed and downloaded by users (e.g., land managers) as needed on smartphones via internet. Then land managers are able to fill the questionnaires and upload the results to the data center within a relatively short period.

## 4. Potentials for Improving ALS Studies

**Figure 6:**
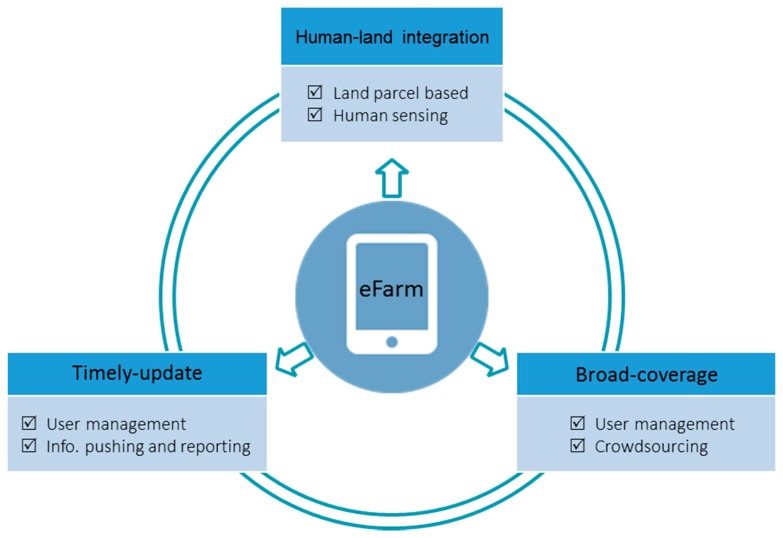
Solutions from eFarm for improving the observation of ALS.

The understandings on our living environment will be greatly improved if its biophysical and socioeconomic features can be better sensed in an integrated manner [40]. eFarm was developed following this idea that it is able to acquire the human–land integrated information which is broadly-covered and updated timely (Figure 6). It has great potential for improving the observation as well as the subsequent analysis on ALS.

### 4.1. Advanced Data Sensing System for Agriculture

The eFarm app can be used as a unified sensing tool to observe the timely information from the field, in terms of crop cover, crop growth etc., which would be able to provide abundant and well-managed ground truth for validating and thus improving the image-based land cover mapping and agricultural remote sensing. In turn, it would also help to promote data fusion, transmission, computation, and explanation ability of traditional agricultural remote sensing. At the same time, it can be used as an open social survey tool for broader purposes to collect household characteristics and their land use activities as complementary information to the identified land parcels (see Section 3.6).

In addition to providing the ground truth, eFarm is able to innovate the traditional image-based remote sensing from a human-centric perspective. For example, it could be applied for disaster monitoring, as challenges still exist in using satellite imagery, especially for damage assessment and diagnosis [41,42]. It could be done in a new way: farmers voluntarily report the damage in their fields with detailed information of location, real-time photos, and descriptions. After diagnosing at the desktop system interacting with other supportive data as well as expert knowledge, the treatment suggestions will be available immediately through the information pushing channel. This information pushing mechanism could also be applied for early-warning before damage actually happens or for advanced production predictions.

### 4.2. Advanced Land Systems Mapping, Modeling, and Comparison

Although remote sensing and spatial modelling have transformed the way we observe global land-use patterns, anthropogenic systems are not directly observable from space and cannot be modelled without a grasp of how humans interact with the environment locally [43]. The improved observation will further advance the mapping and modeling studies of ALS. For example, the land use intensity and field size was difficult to be monitored with the traditional measurements [44,45,46], while the mapping of agricultural intensification could be substantially improved with the support of the detailed human–land integrated data. It is able to provide information on the fragmentation of cropland (represented by the size of the land parcels), the scale of farming (represented by the total land area managed by each land manager), as well as the input-output of the ALS.

**Figure 7:**
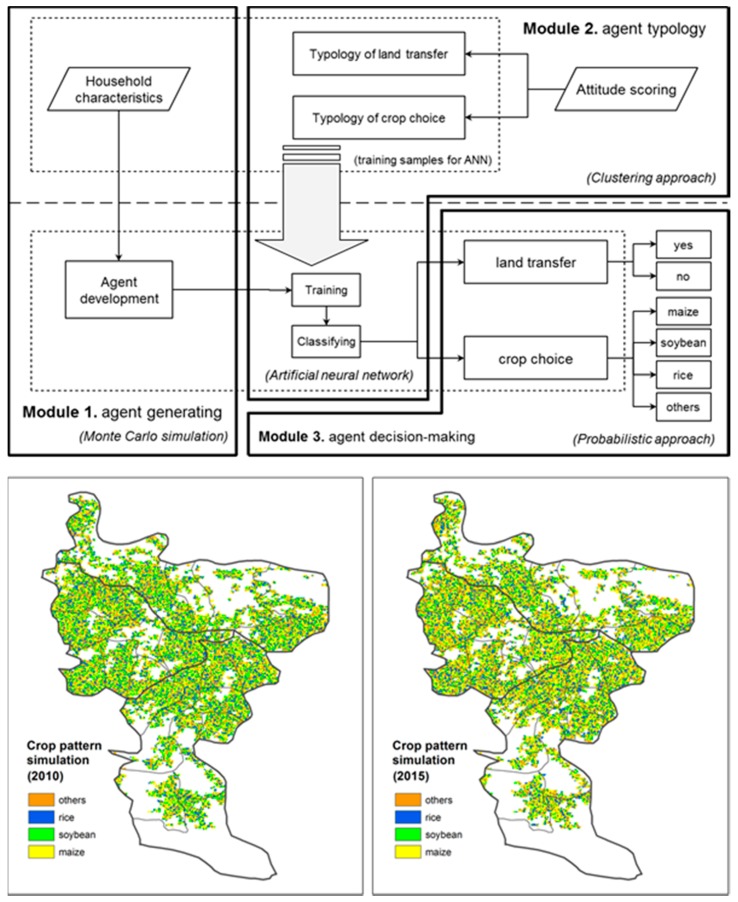
Application of the CroPaDy model based on the human–land integrated information.

Moreover, the detailed and geotagged land use information sensed from individual farmers would facilitate advanced ALS studies, such as agent-based land system modeling [17], by linking together the different research perspectives (micro and macro respectively) and the different aspects of datasets (human-sensing and remote sensing, respectively) (Table 1). At such an initial development stage, a well conceptualized agent-based land system change model called CroPaDy [47] has been linked with the database of eFarm, which enables a direct modeling work given the required data such as household characteristics and land use decisions are available (Figure 7).

It is worthy to notice that the heterogeneities across regions due to the site-specific characteristics of ALSs [48]. However, the traditional household surveys are somehow impossible for large-scale application of cross-site comparison because of its high cost during data collection. Applying the unified and timely data collecting procedure in different regions from various users would help to extend the coverage of data collection thus getting more insights from the analysis, which are always difficult to be carried out with traditional measurements such as household surveys [49,50]. With the help of the crowdsourcing technique, it will have great potential to compare local scale studies across regions.

## 5. Discussion and Conclusions

Humans are low-cost, effective sensors that could potentially contribute to scientific research [33,40]. While great progress has been made in smartphone sensing and VGI by regarding humans as sensors, the application of such sensing techniques are inadequate in ALS studies. We present a new smartphone-based tool to crowdsource the human–land integrated information from a human-centric perspective, in combining the biophysical land parcel information observed from traditional agricultural remote sensing. The main functionalities of the app include visualization of maps, data management of land parcels and users, and data sensing of land and household information.

The guaranteed data quality is always concerned by data users and most people believe that the credibility of those public voluntary contributions is crucial [36,51]. A quite successful data controlling procedure is to let the entries be, to some extent, monitored by volunteers and be open to public editing [52,53]. This can be applied to the current system, for example, the information provided by land managers to be checked with information provided by volunteers, and the sequence of reliability of different users is specified in the previous text (e.g., Section 3.5). Moreover, given its close relationship with SAGI, a more reliable and objective measurement can be applied: to let the crowdsourced information cross-checked with the results of agricultural remote sensing. For example, the reliability of crowdsourced information will be low if there is inconsistency found between crowdsourced information and remote-sensed information. However, due to all of the experiment data being deliberately acquired for system development and the large-scale and long-term crowdsourcing application not yet being available, the accuracy-checking practice will have limited significance at the moment. The more advanced data accuracy-checking approaches or even specialized workers [54] are supposed to be involved for eFarm in the future, especially when it is applied for operational use.

The implementation and adoption of modern technologies always faces challenges and barriers outside of the technologies themselves [55]. For example, incentives are a big issue for persuading people to be involved into the citizen science projects [33,36]. Why is it that volunteers who have no obvious incentive are nevertheless willing to spend large amounts of time creating the layers and content of land parcels? Goodchild [33] stated that self-promotion is the key for VGI, however, it may not be applicable in the current case, especially given the majority of farmers commonly receives less education. This is associated with another issue: the ethics of big data in big agriculture. There is increasing concern about how the use of big data could be more equitable, given the major power is shifting from farmers to corporations in the current era of industrial agriculture. An example is Monsanto, who is also crowdsourcing data from agricultural field [56]. The best solution might be sharing information based on analysis to the data provider in turn, while the open source data remains anonymized to prevent the deleterious exploitation of data as well. Moreover, while crowdsourcing is a promising approach to share knowledge and observe the world at a large scale and with relatively low cost, it also raises some critical security and privacy issues that impede the application [57]. Although the attempts to pollute user contributed data have been rare, but this seems unlikely to remain true for long [58]. Since eFarm wants to acquire and store tracking data about the users and their work in the field, security and privacy challenges need to be appropriately addressed, because the same holds for the acquisition of the household data that can be considered personal data. As a result, how to build an effective information feedback and secured service system for the volunteers is as equally important as the sensing technique itself, but it is apparently out of the scope of this paper.

eFarm is designed to support the human-land integrated data sensing at a county-level basis, while information can be exchanged for regional level applications. It means the system should be able to, at least, cover all the cropland parcels in a county and to store/visualize all the basemaps, created data management units, and sensed land use information in an independent database. The workload of a county level database is about 2.31 terabyte (based on the trial data, details can be found from Supplementary Materials). A successful operational running system not only requires a well-designed framework, and a few essential technologies, but it also needs sound maintenance, timely updates, and well-established communications between data users and data providers. The authors’ host institute (IARRP, CAAS) has been successfully running a national level agricultural remote sensing monitoring system over 20 years [59,60]. It is committed in the next decade to upgrade the existing system into the “big data system of smart farming” by integrating various technologies and data sources together, while the development, application, and maintenance of eFarm are the priorities. Although the large-scale and long-term crowdsourcing application has not been carried out yet, the results of the trial test suggest the system works well, which shows the potential in promoting the sensing, mapping, and modeling of ALS studies. Moreover, the current available version of the app is developed for tablet use only, considering the feasibility of use—e.g., farmers/volunteers may not always carry a tablet—the development and tests of small screen cellphone fitted app versions will be carried out subsequently. In addition to this generalized introductory paper, the application of the tool and results of detailed case studies will be published gradually. We welcome comments and suggestions from the community to co-design and co-apply the tool, not only for improving the tool itself, but also for the broad applications in ALS studies.

## Figures and Tables

**Table 1:** Advanced ALS studies by integrating deferent research perspectives.

Micro Perspective (Actor-Based)	Macro Perspective (Spatial Map-Based)
Land transfer	Agricultural enlargement
Crop choice	Crop pattern
Farm management	Agricultural intensification
Crop yield	Food production

## Supplementary Materials

The following are available online at http://www.mdpi.com/1424-8220/17/3/453/s1. Figure S1: Estimated workload for a county level database, Figure S2: A color-blinded figure describing the overview of the eFarm system, Table S1: Estimated coverage of cropland area, number of land parcels, and number of farmer household at an average level for each county, Table S2: Estimated data volume of basemaps (unit: byte), Table S3: Estimated data volume of created household and land parcel (unit: byte), Table S4: Estimated data volume of sensed land use information (unit: byte).
