# Supplementary material for: eFarm: A Tool for Better Observing Agricultural Land Systems
Source: Sensors (Basel). 2017 Feb 24;17(3):453. doi: 10.3390/s17030453 (PMC5375739; doi:10.3390/s17030453)
Supplement: Supplementary file 1 [file sensors-17-00453-s001.pdf]

Supporting Information for “eFarm: a tool for better observing agricultural land systems”

Estimation of the average workload for a county level database

eFarm is designed to support the human-land integrated data sensing at a county-level basis. According to the existing studies and statistics, we estimated the coverage of cropland area, number of land parcels, and number of farmer household at an average level for each county (Table S1).

Table S1. Estimated coverage of cropland area, number of land parcels, and number of farmer household at an average level for each county

	Cropland area (km2)	Land parcels	Farmer households
Per square kilometer	1	500	150
Per county	800	400,000	120,000

The database workload mainly consists by three parts: the basemaps, the created households and land parcels (i.e. data management unit) and the land use information on each land parcels. The estimated workload of these aspects are presented in Table S2, S3, and S4. All the estimations are based on the supporting trial data acquired for system development.

Table S2. Estimated data volume of basemaps (unit: byte)

Basemap selection	Resolution (m)	Data volume (per 1 km2)	Coverage (km2)	Data volume
Google Map	ca. 0.3	100,000,000	800	80,000,000,000
UAV	ca. 0.1	2,000,000,000	800	1,600,000,000,000

Table S3. Estimated data volume of created household and land parcel (unit: byte)

Data class	Number of field	Field size (average)	Record (per 1km2)	Data volume (per 1 km2)	Coverage (km2)	Data volume
Household	60	765	150	6,885,000	800	5,508,000,000
Land parcel	35	450	500	7,875,000	800	6,300,000,000

Table S4. Estimated data volume of sensed land use information (unit: byte).

Data class	Number of field	Field size	Record (per year)	Number of land parcels	Record (per county)	Data volume
Crop choice	15	765	5	400,000	2,000,000	22,950,000,000
Management	10	765	20	400,000	8,000,000	61,200,000,000
Pictures	1	51,200	30	400,000	12,000,000	614,400,000,000

The estimated total workload for a county level database is presented in Figure S-1. The upper and lower whiskers are the variation caused by the use of basemaps. The upper workload boundary is about 2.31 TB (terabyte), if all the basemaps are from UAV images. Given most of the places can be covered with high resolution GE images, the actual workload will be lower than this. If 80% basemaps are supported by GE (while the rest are obtained by UAV), the workload would be 1.1 TB (Figure S1).

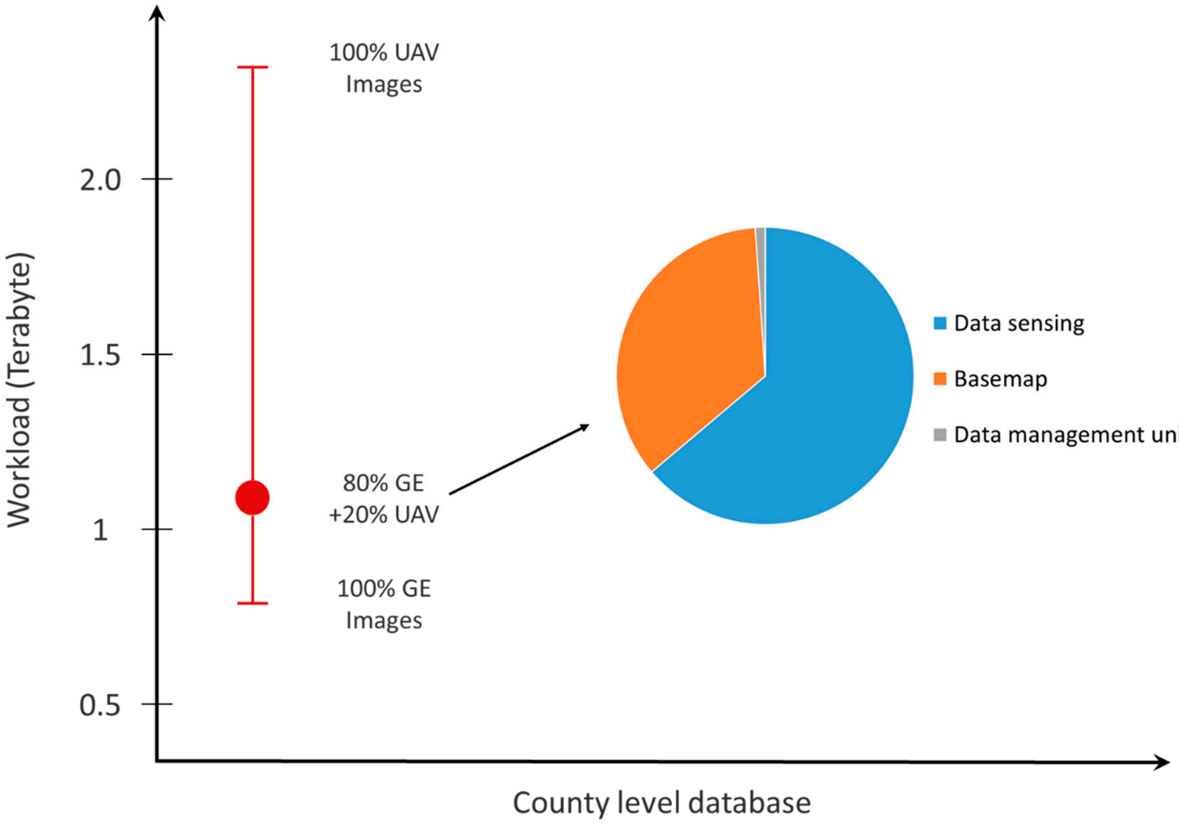

Figure S1. Estimated workload for a county level database.

The overview of the eFarm system.

A color-blinded figure describing the overview of the eFarm system is presented in Figure S2, in case that readers may print out the paper in black and white. The light-gray (orange in the main document) labeled section illustrates how the smartphone app works; the mid-dark-gray (blue in the main document) labeled section illustrates how data is exchanged among different platforms; and the dark-gray (green in the main document) labeled section shows the how the comprehensive ALS information will be applied for sophisticated analysis.

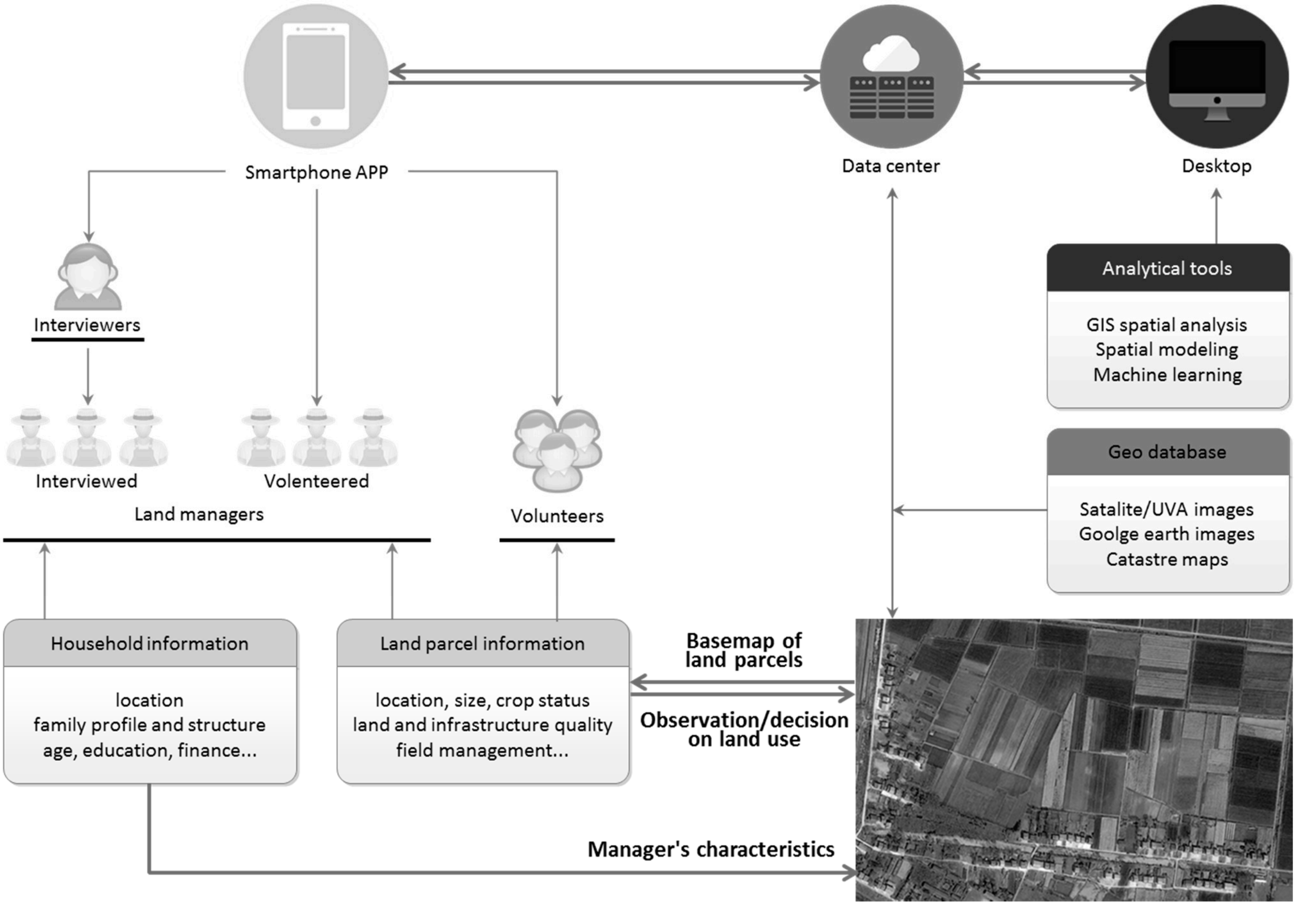

Figure S2. A color-blinded figure describing the overview of the eFarm system.
